# Plant use in Odo-Bulu and Demaro, Bale region, Ethiopia

**DOI:** 10.1186/1746-4269-7-28

**Published:** 2011-09-24

**Authors:** Rainer W Bussmann, Paul Swartzinsky, Aserat Worede, Paul Evangelista

**Affiliations:** 1 William L. Brown Center, Missouri Botanical Garden, P.O. Box 299, St. Louis, MO 63166-0299, USA; 2 The Murulle Foundaion, PO Box 1442, Fort Collins, CO 80522, USA; 3 Rift Valley Safaris, PO Box 3658 Addis Ababa, Ethiopia; 4 Natural Resource Ecology Laboratory, Colorado State University, Fort Collins, CO 80523-1499, USA

**Keywords:** Oromo, Ethiopia, Ethnobotany, Plant use, traditional knowledge, utilization

## Abstract

This paper reports on the plant use of laypeople of the Oromo in Southern Ethiopia. The Oromo in Bale had names/uses for 294 species in comparison to 230 species documented in the lower reaches of the Bale area. Only 13 species was used for veterinary purposes, or as human medicine (46). Plant medicine served mostly to treat common everyday ailments such as stomach problems and diarrhea, for wound treatment and as toothbrush-sticks, as anthelmintic, for skin infections and to treat sore muscles and. Interestingly, 9 species were used to treat spiritual ailments and to expel demons. In most cases of medicinal applications the leaves or roots were employed.

Traditional plant knowledge has clearly declined in a large part of the research area. Western style health care services as provided by governments and NGOs, in particular in rural areas, seem to have contributed to a decline in traditional knowledge, in part because the local population simply regards western medicine as more effective and safer.

## Introduction

Plants have been an integral part of life in many indigenous communities, and Africa is no exception [1,2]. Apart from providing building materials, fodder, weapons and other commodities, plants are especially important as traditional medicines. Many tribes and cultures in Africa have an elaborated plant knowledge-base [3]. Most of this knowledge is still entirely transferred orally within the family unit or community [4]. Western influences have, however, led to an accelerating decline of this tradition. For example, Western style healthcare supplied by some governments has been expanded in the last decades, but it is still often not readily available and many regions remain completely underserved. Subsequently, most rural communities still use herbal remedies as readily and cheaply available alternatives. This knowledge is however, rapidly dwindling due to desired changes towards a more Western lifestyle, and the influence of modern tourism and other agents of globalization.

During the last decades, a vast array of ethnobotanical studies from Ethiopia has been published. Most of these focused however on the northern regions [5-12], as well central and southern Ethiopia [13-26].

Various studies report on the toxicity and efficacy of Ethiopian traditional medicine [8,27-34].

### The study area

**Figure 1:**
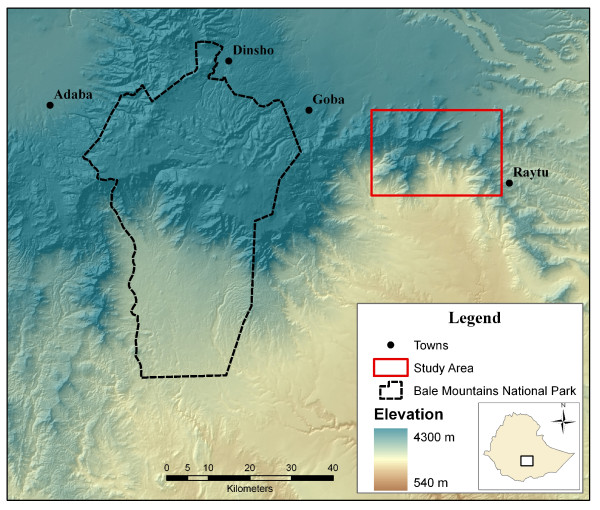
**Study area**.

Our study was conducted in the eastern reaches of the Bale Mountains in the southern highlands of Ethiopia (approximately 6° 9'N, 40° 22'W) [35]. The study area covers an area approximately 380 km^2 ^with elevations ranging from 1,500 m to 3,300 m (Figure 1). Mean minimum and maximum temperatures are 10.2 C° and 21.3 C°, respectively; while mean annual precipitation ranges from 68 to 93 mm largely occurring during two rainy seasons. The majority of the study area is mountainous with intact forest ecosystems [36,37]. Most anthropogenic activities are centered on honey gathering and the collection of wood and bamboo (*Sinarundinaria alpina*). Some livestock grazing occurs, but generally at small scales. The study area has remained relatively preserved for two primary reasons: the topography is largely prohibitive to cultivation and there are two controlled hunting concessions (called Odu Bulu and Demaro) that provide legal protection to the forest. Trophy hunting within the hunting concessions generally occurs within a three-month period; however, both concessions maintain permanent camps and guards to protect the wildlife and habitat. Just beyond the northern edges of the study area, the landscape is heavily populated with people and livestock. The forests here have long been cleared, and barley cultivation is extensive. The southern edge of the study area drops sharply in elevation before transforming into semi-arid plains that stretch into Somalia. The steep slopes act as a barrier to human and livestock encroachment providing further protection to the study area [38]. Although the study area has significantly less anthropogenic impact than nearby Bale Mountains National Park, increasing human and livestock pressure within the study area is becoming evident.

### The Oromo

The Oromo are the main ethnic group in southern Ethiopia, including the Bale region, although members of many other peoples have settled in the area. Smaller populations are found in Somalia and northern Kenya. Barley and wheat cultivation provide most sustenance and income in Bale, with some areas receiving enough rainfall to support two harvests a year. Livestock keeping is also important to Oromo people, but occurs to a lesser extent than most areas in Ethiopia. During the time when crops are cultivated, livestock are grazed in the forest and Afro-alpine of higher elevations. Because some areas can support two harvests annually, livestock may spend as long as ten months in natural areas. During the last decades, Bale has seen profound changes, from increased access and governmental health care entering during the communist era of the 1970s and 80s, to an increase in tourism in the 1990s and a large influx of Chinese development aid in the last few years. These years have also marked a dramatic increase in human and livestock populations, and consequently land-use and conversion of the landscape. Throughout the 1980s and early 90s, Ethiopia's communist government regularly relocated people from northern regions to Bale as a means to disrupt civil opposition [39]. Since then, the current government has continued the practice on a voluntary basis as an effort to provide people access to natural resources, which have been depleted in other parts of the country. Collectively, these events have put an enormous strain on forests in the Bale Mountains, and are changing the local economy and traditional customs profoundly.

The ethnobotany of various subgroups of the Oromo has been focal point of a few recent studies [40-42].

## Materials and Methods

### Ethnobotanical data and plant collections

Fieldwork was carried out between 2009 by Bussmann and collaborators. To obtain information on plants used traditionally, interviews were conducted using semi-structured questionnaires [43]. Random sampling technique was applied in distributing the questionnaires. Before carrying out the interviews, an oral prior informed consent was sought from every respondent. All communities involved showed the same acceptance of the researchers, and similar in-field times were involved in the study in order to avoid possible errors in data depth.

A total of 12 lay respondents were interviewed. Access to female informants was not possible. In order to get a more detailed inventory of plant use, ethnobotanical data were collected by conducting interviews directly in the field during collection trips, and by discussing the freshly collected specimens with informants, after seeking oral consent from each respondent. This method was preferred over pure questionnaires to also get an indication for species that are not used by the community, and which are normally not mentioned during traditional interviews. All interviews were carried out in local language by native speakers, and then translated into English. Voucher specimens were collected and are preserved at the National Herbarium of Ethiopia (ADD). The identification of plant material followed the Flora of Ethiopia and Erithrea [44-50], as well as [51-53]. Plant nomenclature follows TROPICOS http://www.tropicos.org.

## Results and Discussion

**Figure 2:**
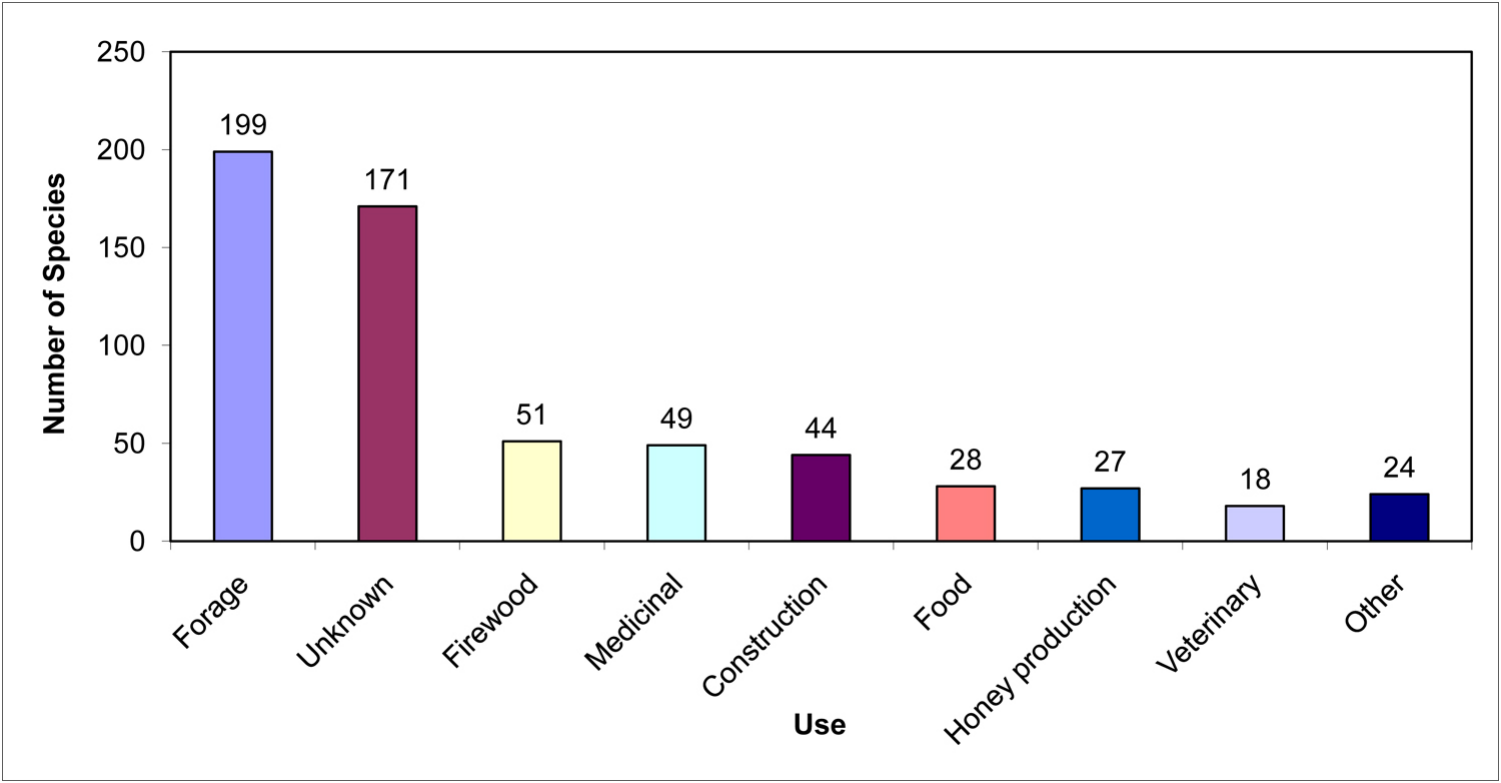
**Number of plant species used in each use category**.

The Oromo in Bale had names/uses for 294 species encountered (Table 1.), in comparison to 230 species documented in the lower reaches of the Bale area [23], and 101 species in the highlands [41]. The latter study did however interview health experts, while the present work focused on the knowledge of laypeople. One hundred and sixty two species encountered in this study were classified as having no uses whatsoever, although many of them were named. Many of the identified species had multiple uses or were known provide important direct or indirect services to the community (Figure 2). Most species named (172 species) were used for livestock grazing (mostly cattle). The vernacular name "Marga" for many Poaceae simply translates to "grass", and underlines the importance of this resource. It is important to note however that 42 of these were also indicated to be important for the endemic and endangered mountain nyala (*Tragelaphus buxtoni*), illustrating a potential conflict between pastoralist use and wildlife conservation. A further 27 species were used as fodder for both domestic animals and eaten by wildlife. Again the vernacular names often pointed to that specific use. *Argemone mexicana *and similar spiny species were all called "Korehare" which translates to "spiny donkey", and all serve as fodder for donkeys. Nine species were used as poisons against carnivores. Fifty-one tree species were used as firewood, while only two served to produce charcoal. Traditional houses are to a large extent built using material from the forest, and it is not astonishing that 15 tree species were used for timber, 17 species provided material to make ropes, mostly used to tie the house posts and roof beams, and 10 species were used as thatch. A wide variety of plants was found to be employed for the fabrication of tools and household implements (3 for brooms, 4 to make beehives, 3 for tanning, 11 to make ploughs, 2 served as detergent to wash clothes). In addition forest species were an important source of nutrients, with 28 species collected as food, and 23 explicitly used for honey production.

**Table 1:** Plants encountered in Odo Bulu and Demaro, Bale, Ethiopia

Voucher	Oromifa	Family	Scientific name	Uses and notes
16191	Sokoro	Acanthaceae	*Acanthus *sp.	No use.
16011	Sokoro	Acanthaceae	*Acanthus eminens *C.B. Clarke	Flowers for honey.
16011	Sokoro	Acanthaceae	*Acanthus eminens *C.B. Clarke	NOT eaten by animals.
16011	Sokoro	Acanthaceae	*Acanthus sennii *Chiov.	Medicine; leaves are dried, ground, mixed with butter and applied to wounds.
16236	Sokoru	Acanthaceae	*Acanthus sennii *Chiov.	Flowers for honey.
16223	Dergu	Acanthaceae	*Dicliptera laxata *C.B. Clarke	Eaten by cattle and mountain nyala. NOTE: "Flower is different from place to place".
16210	Dergu	Acanthaceae	*Dicliptera *sp.	Eaten by cattle.
16210	Dergu	Acanthaceae	*Dicliptera *sp.	Flowers for honey.
15999	Dergu	Acanthaceae	*Hypoestes forskaolii *(Vahl.) R. Br.	Eaten by animals.
16293	Gurbi	Acanthaceae	*Hypoestes *sp.	Eaten by cattle and mountain nyala.
16220	Dergu	Acanthaceae	*Hypoestes triflora *(Forssk.) Roem. & Schult.	Eaten by cattle and mountain nyala.
16046	Dergu	Acanthaceae	*Justicia diclipteroides *Lindau	Eaten by cattle, bushbuck and mountain nyala.
16263	Gurbi	Acanthaceae	*Justicia diclipteroides *Lindau	Eaten by cattle.
16336	Umuga	Acanthaceae	*Justicia schimperiana *(Hochst. Ex Nees) T. Anderson	Rope; bark peeled and used as rope for construction to attach the wall fragments.
16288	Gurbi	Acanthaceae	*Justicia *sp.	Eaten by cattle and mountain nyala.
16040	Herraye	Acanthaceae	*Minulopsis solmsii *Schweinf.	Flowers for honey. NOTE: "This flowers only once in seven years. I have seen it twice in my lifetime (the speaker was about 55 years old). The last flowering was about 10 years ago when the great rain stopped. (El Niño 1998). It also flowered before the king was replaced (around 1973).
16237	Anano	Acanthaceae	*Thunbergia alata *Bojer ex Sims	Eaten by cattle and mountain nyala.
16294	Gurbi	Acanthaceae		Eaten by cattle and mountain nyala.
16313	Gurbi	Acanthaceae		Eaten by cattle and mountain nyala.
16397	No name	Acanthaceae		No use.
16408	No name	Acanthaceae		No use.
16228	Hacho	Amaranthaceae	*Achyranthes aspera *L.	Eaten by cattle.
16228	Hacho	Amaranthaceae	*Achyranthes aspera *L.	Veterinary; crush the root, boil it in water and give the animals to drink against rabies.
16144	Rafu	Amaranthaceae	*Amaranthus *sp.	Eaten by cattle.
16144	Rafu	Amaranthaceae	*Amaranthus *sp.	Food; cooked like cabbage in time of drought.
16153	Ch'okene	Amaranthaceae	*Amaranthus *sp.	Medicine; as remedy for spiritual pain. Inhaling the smell brings out the evil. NOTE: Name translates to "tall".
16153	Ch'okene	Amaranthaceae	*Amaranthus *sp.	NOT eaten by livestock.
16153	Ch'okene	Amaranthaceae	*Amaranthus *sp.	Veterinary; crushed and smeared on cattle for spiritual protection and "highsight".
16355	Rafu	Amaranthaceae	*Amaranthus *sp.	Eaten by cattle.
16379	Rafu	Amaranthaceae	*Amaranthus *sp.	No use.
16303	Gurbi	Amaranthaceae	*Celosia anthelminthica *Aschers.	Eaten by cattle and mountain nyala.
16007	Hacho	Amaranthaceae	*Cyathula cylindrica *Moq.	Eaten by cattle.
16007	Hacho	Amaranthaceae	*Cyathula cylindrica *Moq.	Veterinary; crush the root, boil it in water and give the animals to drink against rabies.
16247	Hacho	Amaranthaceae	*Cyathula polycephala *Baker	Eaten by cattle.
16127	Hacho	Amaranthaceae	*Cyathula uncinulata *(Schrad.) Schinz	Eaten by cattle.
16127	Hacho	Amaranthaceae	*Cyathula uncinulata *(Schrad.) Schinz	Veterinary; crush the root, boil it in water and give the animals to drink against rabies.
16216	Hacho	Amaranthaceae	*Cyathula uncinulata *(Schrad.) Schinz	Eaten by cattle.
16216	Hacho	Amaranthaceae	*Cyathula uncinulata *(Schrad.) Schinz	Veterinary; crush the root, boil it in water and give the animals to drink against rabies.
16297	No name	Amaranthaceae		No use.
16376	Komudu	Amaranthaceae		No use.
16289	Tadesa	Anacardiaceae	*Rhus *sp.	Eaten by cattle and mountain nyala.
16289	Tadesa	Anacardiaceae	*Rhus *sp.	Food; children eat the sweet fruits.
16213	No name	Apiaceae	*Agrocharis incognita *(C. Norman) Heyw. & Jury	Eaten by cattle.
16213	No name	Apiaceae	*Agrocharis incognita *(C. Norman) Heyw. & Jury	Medicine; root is crushed and eaten for stomach problems.
16213	No name	Apiaceae	*Agrocharis incognita *(C. Norman) Heyw. & Jury	Veterinary; root is crushed and given to livestock for stomach problems.
15986	No name	Apiaceae	*Carum *sp.	No use.
16182	No name	Apiaceae	*Hydrocotyle mannii *Hook.f.	Eaten by baboons.
16010	Informant does not remember name	Apiaceae	*Pimpinella oreophila *Hook. f.	Eaten by livestock and wildlife.
16010	Informant does not remember name	Apiaceae	*Pimpinella oreophila *Hook. f.	Medicine; roots are ground and prepared as tea for stomach problems.
15987	No name	Apiaceae	*Sanicula elata *Buch.-Ham. ex D. Don.	No use.
15993	Informant does not remember name	Apiaceae	*Torilis arvensis *(Huds.) Link	Eaten by animals.
16088	No name	Apiaceae		No use.
16115	No name	Apiaceae		No use.
16171	No name	Apiaceae		Eaten by baboons.
16326	Hagamsa	Apocynaceae	*Carissa edulis *(Forssk.) Vahl	Eaten by goats.
16326	Hagamsa	Apocynaceae	*Carissa edulis *(Forssk.) Vahl	Food; fruit eaten by humans.
16027	Homba	Apocynaceae	*Oncinotis tenuiloba *Stapf.	Eaten by livestock and mountain nyala. NOTE: The latex is very sticky but not poisonous.
16027	Homba	Apocynaceae	*Oncinotis tenuiloba *Stapf.	Rope. NOTE: The latex is very sticky but not poisonous.
16423	Bulala	Apocynaceae	*Oncinotis tenuiloba *Stapf.	Rope for construction.
16330	Diki	Apocynaceae		Eaten by cattle and mountain nyala (leaves).
16330	Diki	Apocynaceae		Rope; bark peeled and used as rope for construction.
16333	Gidila	Apocynaceae		Poison; used to kill carnivores (mainly hyenas and lions); dry plant, crush and put on meat.
16400	Anano	Apocynaceae		Eaten by cattle and mountain nyala.
16422	Anano	Apocynaceae		Eaten by cattle and mountain nyala.
16031	Abeye	Aquifoliaceae	*Ilex mitis *(L.) Radkl.	Food; women use the leaves to roll the dough in before putting it in the oven so that it does not burn. The seeds are crused and the oil is used to grease the baking plate before baking.
16414	Arfatu	Araliaceae	*Cussonia holstii *Harms ex Engl.	Beehives (wood).
16214	Gatami	Araliaceae	*Schefflera abyssinica *(Hochst. ex Rich.) Harms	Firewood.
16214	Gatami	Araliaceae	*Schefflera abyssinica *(Hochst. ex Rich.) Harms	Flowers for honey.
16214	Gatami	Araliaceae	*Schefflera abyssinica *(Hochst. ex Rich.) Harms	NOT eaten by cattle.
16025	Ansha	Araliaceae	*Schefflera volkensii *(Harms) Harms	Eaten by cattle and colobus monkeys.
16025	Ansha	Araliaceae	*Schefflera volkensii *(Harms) Harms	Firewood.
16041	Ansha	Araliaceae	*Schefflera volkensii *(Harms) Harms	Firewood.
16043	Meti	Arecaceae	*Phoenix reclinata *Jacq.	Weave wedding baskets and floor mats.
16134	Seriti	Asparagaceae	*Asparagus africanus *Lam.	Eaten by cattle and wildlife.
16134	Seriti	Asparagaceae	*Asparagus africanus *Lam.	Medicine; crush the plant, extract the juice and put on pimples.
16337	Sariti	Asparagaceae	*Asparagus africanus *Lam.	Making brooms.
16135	Seriti	Asparagaceae	*Asparagus falcatus *L.	Eaten by cattle and wildlife.
16135	Seriti	Asparagaceae	*Asparagus falcatus *L.	Medicine; crush the plant, extract the juice and put on pimples.
16202	No name	Asparagaceae	*Chlorophytum ducis-aprutii *Chiov.	No use.
15990	Kokosa	Aspleniaceae	*Asplenium friesiorum *C. Chr.	No use.
16017	Kokosa	Aspleniaceae	*Asplenium monanthes *L.	Sometimes eaten by cattle and mountain nyala.
16013	Kokosa	Aspleniaceae	*Asplenium *sp.	Sometimes eaten by cattle and mountain nyala.
16014	Kokosa	Aspleniaceae	*Asplenium *sp.	Sometimes eaten by cattle and mountain nyala.
16099	Kokosa	Aspleniaceae	*Asplenium *sp.	No use.
16120	Kokosa	Aspleniaceae	*Asplenium *sp.	No use.
16196	Kokosa	Aspleniaceae	*Asplenium *sp.	No use.
16197	Kokosa	Aspleniaceae	*Asplenium *sp.	No use.
16198	Kokosa	Aspleniaceae	*Asplenium *sp.	No use.
16206	No name	Aspleniaceae	*Asplenium *sp.	No use.
16207	Kokosa	Aspleniaceae	*Asplenium *sp.	No use.
16012	Kokosa	Aspleniaceae	*Asplenium theciferum *(Kunth.) Mett.	Sometimes eaten by cattle and mountain nyala.
16244	Gurbi	Asteraceae	*Ageratum conyzoides *L.	No use.
16382	Kore'apata	Asteraceae	*Arctium lappa *L.	No use.
16147	Ch'okone	Asteraceae	Artemisia absinthium (Mill.) DC.	Medicine; as remedy for spiritual pain. Inhaling the smell brings out the evil. NOTE: Name translates to "tall".
16147	Ch'okone	Asteraceae	Artemisia absinthium (Mill.) DC.	NOT eaten by livestock. NOTE: Name translates to "tall".
16147	Ch'okone	Asteraceae	Artemisia absinthium (Mill.) DC.	Veterinary; crushed and smeared on cattle for spiritual protection and "highsight". NOTE: Name translates to "tall".
16354	Informant does not remember name	Asteraceae	Artemisia absinthium (Mill.) DC.	No use.
16113	Hada	Asteraceae	*Bidens *sp.	Eaten by cattle.
16190	Korehare	Asteraceae	*Carduus nyassanus *(S. Moore) R.E. Fr.	Eaten by donkeys. NOTE: Name translates to "spiny donkey".
16125	No name	Asteraceae	*Centaurea *sp.	No use.
16039	No name	Asteraceae	*Cineraria deltoidea *Sond.	Eaten by cattle.
16122	Korehare	Asteraceae	*Cirsium dender *Friis	Eaten by donkeys. NOTE: Name translates to "spiny donkey".
16368	Korehare	Asteraceae	*Cirsium vulgare *(Savi) Ten.	Eaten by donkeys. They prefer the heads. NOTE: Name translates to "spiny donkey".
15998	Informant does not remember name	Asteraceae	*Crassocephalum *sp.	Eaten by cattle.
16038	No name	Asteraceae	*Crassocephalum *sp.	Eaten by cattle.
16409	No name	Asteraceae	*Crepis *cf. *rueppellii *Sch. Bip.	No use.
16052	Korehare	Asteraceae	*Echinops hoehnelii *Schweinf.	Eaten by donkeys.
16204	No name	Asteraceae	*Galinsoga parviflora *Cav.	No use.
16108	No name	Asteraceae	Gnaphalium sp.	Eaten by cattle.
16145	Informant does not remember name	Asteraceae	*Haplocarpha rueppellii *(Sch. Bip.) K. Lewin	Eaten by cattle.
16176	No name	Asteraceae	*Helichrysum formosissimum *Sch. Bip.	No use.
16093	No name	Asteraceae	*Helichrysum *sp.	No use.
16094	No name	Asteraceae	*Helichrysum *sp.	No use.
16142	No name	Asteraceae	*Helichrysum *sp.	No use.
16357	Hariti	Asteraceae	*Helichrysum *sp.	No use.
16055	Hatawi	Asteraceae	*Inula confertiflora *A. Rich.	Poison; this is NOT eaten by animals. The leaves look like nice toilet paper but should not be used because they will cause swellings.
15988	No name	Asteraceae	*Mikania *sp.	Eaten by cattle.
16002	Karkora	Asteraceae	*Mikaniopsis clematoides *Milne-Redh.	Eaten by cattle and mountain nyala.
16205	No name	Asteraceae	*Mikaniopsis *sp.	Medicine;. "eye medicine" for better spiritual view (chewed root). The root is also chewed to protect against Evil Eye.
16161	Anono	Asteraceae	*Prenanthes subpeltata *Stebbins	Medicine; leaves are boiled and then put on swellings and bruises.
16165	Anono	Asteraceae	*Prenanthes subpeltata *Stebbins	Medicine; leaves are boiled and then put on swellings and bruises.
16037	Hagedena	Asteraceae	*Senecio *sp.	Flowers for honey.
16089	No name	Asteraceae	*Senecio *sp.	Eaten by cattle.
16095	Adado	Asteraceae	*Senecio *sp.	Rope; to tie the main pole of the house. Very durable.
16095	Adado	Asteraceae	*Senecio *sp.	Firewood.
16114	No name	Asteraceae	*Senecio *sp.	No use.
16174	No name	Asteraceae	*Senecio *sp.	No use.
16175	No name	Asteraceae	*Senecio *sp.	No use.
16430	Buritaro	Asteraceae	*Senecio *sp.	No use.
16131	Rafu	Asteraceae	*Solanecio angulatus *(Vahl) C. Jeffrey	Medicine; the leaves are boild and the steam inhaled for spiritual cleansing and to expel spirits in crazy people.
16131	Rafu	Asteraceae	*Solanecio angulatus *(Vahl) C. Jeffrey	NOT eaten by cattle.
16132	Galesimbira	Asteraceae	*Sonchus bipontini *Asch.	Eaten by cattle (given to calves to strengthen them). NOTE: Name translates to "birdvine".
16132	Galesimbira	Asteraceae	*Sonchus bipontini *Asch.	Eaten by cattle. NOTE: Name translates to "birdvine".
16132	Galesimbira	Asteraceae	*Sonchus bipontini *Asch.	Medicine; used for swellings. NOTE: Name translates to "birdvine".
16132	Galesimbira	Asteraceae	*Sonchus bipontini *Asch.	Veterinary; used for swellings. NOTE: Name translates to "birdvine".
16166	No name	Asteraceae	*Sonchus oleraceus *L.	No use.
16243	Hada	Asteraceae	*Tagetes erecta *L.	Poison; kills cattle when they eat it.
16243	Hada	Asteraceae	*Tagetes erecta *L.	Poison; very bad for humans. If it gets in a wound it will expand.
16243	Hada	Asteraceae	*Tagetes erecta *L.	Veterinary; used to treat cattle. Crush the leaves and put in the hole a worm made to kill the worms (botfly remedy).
16320	Sojom	Asteraceae	*Vernonia amygdalina *Delile	No use.
16338	Ebicha	Asteraceae	*Vernonia amygdalina *Delile	Construction (timber).
16338	Ebicha	Asteraceae	*Vernonia amygdalina *Delile	Eaten by cattle.
16338	Ebicha	Asteraceae	*Vernonia amygdalina *Delile	Firewood.
16338	Ebicha	Asteraceae	*Vernonia amygdalina *Delile	Veterinary; smash leaves and feed to cattle for stomach problems.
16021	Regi	Asteraceae	*Vernonia *sp.	Flowers for honey.
16021	Regi	Asteraceae	*Vernonia *sp.	Veterinary; the leaves are fed to cattle who have stomach problems to fatten them.
16032	Gadarra	Asteraceae	*Vernonia *sp.	Eaten by cattle.
16053	Kadara	Asteraceae	*Vernonia *sp.	Eaten by cattle and mountain nyala.
16053	Kadara	Asteraceae	*Vernonia *sp.	Poison; the hairs can cause eye problems.
16212	No name	Asteraceae	*Vernonia *sp.	No use.
16230	Hevicha	Asteraceae	*Vernonia *sp.	Eaten by cattle.
16230	Hevicha	Asteraceae	*Vernonia *sp.	Veterinary; crush the leaves and make an extract. Give cattle to drink when they are bloated.
16065	Hadda	Asteraceae		Eaten by cattle.
16091	No name	Asteraceae		No use.
16133	No name	Asteraceae		Flowers for honey.
16133	No name	Asteraceae		NOT eaten by animals.
16168	No name	Asteraceae		No use.
16229	Informant does not remember name	Asteraceae		No use.
16296	No name	Asteraceae		No use.
16328	Sojoma	Asteraceae		No use.
16358	No name	Asteraceae		No use.
16361	Hada	Asteraceae		Eaten by cattle.
16361	Hada	Asteraceae		Flowers for honey.
16377	Anamale	Asteraceae		No use. NOTE: Name translates to "only me" and classifies this as invasive species.
16383	Kore'apata	Asteraceae		Weed.
16384	No name	Asteraceae		No use.
16440	Hada	Asteraceae		Eaten by cattle.
16440	Hada	Asteraceae		Flowers for honey.
16442	No name	Asteraceae		No use.
16104	Ch'afara	Balsamiaceae	*Impatiens tinctoria *A. Rich.	No use.
16000	Tschafara	Balsamiaceae	*Impatiens tinctoria *A. Rich. ssp. *abyssinica *(Hook. f. ex Oliv.) Grey-Wilson	Eaten by porcupine (roots, look like potatos).
16105	Ch'afara	Balsamiaceae	*Impatiens walleriana *Hook. f.	Eaten by pigs, especially the root.
16105	Ch'afara	Balsamiaceae	*Impatiens walleriana *Hook. f.	Medicine; women use the potato like root to strengthen their hair.
16222	No name	Basellaceae	*Basella alba *L.	Eaten by cattle and mountain nyala.
16322	W'odes	Boraginaceae	*Cordia africana *Lam.	Beehives.
16322	W'odes	Boraginaceae	*Cordia africana *Lam.	Construction (timber).
16322	W'odes	Boraginaceae	*Cordia africana *Lam.	Firewood.
16322	W'odes	Boraginaceae	*Cordia africana *Lam.	Food; fruits eaten by baboons and humans.
16162	Korsamichi	Boraginaceae	*Cynoglossum amplifolium *Hochst. ex A. DC.	Medicine; leaves are crushed, mixed with a little water to make an extract, which is drunk or put in the nose to treat colds.
16054	Korichi Michi	Boraginaceae	*Cynoglossum coeruleum *A. DC. ssp. *geometricum *(Baker & C.H. Wright) S. Edwards	Medicine; leaves are crushed and put in water to make an extract. That is applied to cold sores and can also be drunk for stomach problems. NOTE: Name translates to "spiny donkey".
16139	Korichi Michi	Boraginaceae	*Cynoglossum lanceolatum *Forssk.	Medicine; the plant is crushed and applied to cold sores and afts, and also crushed and inhaled for headache.
16139	Korichi Michi	Boraginaceae	*Cynoglossum lanceolatum *Forssk.	NOT eaten by animals.
16143	Informant does not remember name	Boraginaceae	*Lithospermum officinale *L.	Eaten by cattle when flowering. NOTE: "This came with the wheat given for aid during the Derg government".
16150	Carchapa	Boraginaceae	*Myosotis abyssinica *Boiss. & Reut.	Eaten by cattle.
16136	Saro	Brassicaceae	*Brassica *sp.	Eaten by cattle.
16136	Saro	Brassicaceae	*Brassica *sp.	Veterinary; seeds are roasted and crushed and then fed to bloated cattle.
16141	Saro	Brassicaceae	*Brassica *sp.	Eaten by cattle.
16141	Saro	Brassicaceae	*Brassica *sp.	Veterinary; seeds are roasted and crushed and then fed to bloated cattle.
16186	No name	Brassicaceae	*Cardamine hirsuta *L.	No use.
16152	Saro	Brassicaceae		Eaten by cattle.
16152	Saro	Brassicaceae		Veterinary; seeds are roasted and crushed and then fed to bloated cattle.
16241	No name	Brassicaceae		No use.
16398	No name	Burseraceae	*Boswellia rivae *Engl.	No use.
16308	Matakoma	Cabnnabaceae	*Celtis africana *Burm. f.	Firewood.
16308	Matakoma	Cabnnabaceae	*Celtis africana *Burm. f.	Tools; wood used to make ploughs.
16388	Bireliko	Cabnnabaceae	*Celtis gomphophylla *Baker	Firewood.
16419	Matacoma	Cabnnabaceae	*Celtis *sp.	Firewood.
16061	No name	Campanulaceae	*Lobelia *cf *erlangeriana *Engl.	Eaten by baboons.
16073	No name	Campanulaceae	*Wahlenbergia silenoides *Hochst. ex A. Rich	Eaten by cattle.
16441	No name	Campanulaceae	*Wahlenbergia *sp.	No use.
16155	Informant does not remember name	Caryophyllaceae	*Arenaria serpyllifolia *L.	Eaten by cattle.
16155	Informant does not remember name	Caryophyllaceae	*Arenaria serpyllifolia *L.	Medicine; for spiritual things.
16350	Ch'oge	Caryophyllaceae	*Cerastium hirsutum *Crantz	Eaten by cattle. NOTE: Informants specifically mention small flower and fruit.
16110	No name	Caryophyllaceae	*Cerastium indicum *Wight & Arn.	No use.
16238	No name	Caryophyllaceae	*Drymaria cordata *(L.) Willd. ex Roem. & Schult.	Eaten by cattle.
16111	Dukusha	Caryophyllaceae	*Stellaria sennii *Chiov.	To soften leather. Crush the plant and apply to leather to make leather ropes soft.
16077	No name	Caryophyllaceae		Eaten by baboons.
16026	Kombocha	Celastraceae	*Maytenus arbutifolia *R. Wilczek	Eaten by livestock and mountain nyala.
16235	Kombolcha	Celastraceae	*Maytenus arbutifolia *R. Wilczek	Eaten by cattle.
16235	Kombolcha	Celastraceae	*Maytenus arbutifolia *R. Wilczek	Firewood.
16344	Kombolcha	Celastraceae	*Maytenus senegalensis *(Lam.) Exell	Eaten by goats and mountain nyala
16344	Kombolcha	Celastraceae	*Maytenus senegalensis *(Lam.) Exell	Firewood.
16344	Kombolcha	Celastraceae	*Maytenus senegalensis *(Lam.) Exell	NOT eaten by cattle.
16195	Kombolcha	Celastraceae	*Maytenus *sp.	Eaten by cattle and mountain nyala.
16195	Kombolcha	Celastraceae	*Maytenus *sp.	Firewood.
16266	Kombolcha	Celastraceae	*Maytenus *sp.	Eaten by goats.
16266	Kombolcha	Celastraceae	*Maytenus *sp.	Flowers for honey.
16407	Jima	Celastraceae		Firewood.
16146	Bucha	Chenopodiaceae	*Chenopodium *sp.	Eaten by cattle, but if they eat it in the morning they get bloated.
16148	Bucha	Chenopodiaceae	*Chenopodium *sp.	Eaten by cattle, but if they eat it in the morning they get bloated.
16353	No name	Chenopodiaceae	*Chenopodium *sp.	NOT eaten by animals.
16033	Garramba	Clusiaceae	*Hypericum revolutum *Vahl	Construction; timber used for house posts.
16033	Garramba	Clusiaceae	*Hypericum revolutum *Vahl	Medicine; leaves boiled and given to babies with stomach problems and to make babies stop crying.
16076	Sedisa	Clusiaceae	*Hypericum *sp.	Eaten by cattle. NOTE: Informants were not completely sure about the name.
16404	Tantefensa	Combretaceae	*Combretum *sp.	Charcoal.
16404	Tantefensa	Combretaceae	*Combretum *sp.	Firewood.
16209	No name	Commelinaceae	*Commelina imberbis *Ehrenb. ex. Hassk.	No use.
16366	K'aio	Commelinaceae	*Commelina *sp.	Eaten by cattle and mountain nyala.
16393	K'aio	Commelinaceae	*Commelina *sp.	Eaten by cattle.
16078	No name	Commelinaceae	*Cyanotis polyrrhiza *Hochst. ex Hassk.	Eaten by baboons.
16048	Hananu	Convolvulaceae	*Convolvulus kilimandschari *Engl.	Eaten by cattle and especially fed to calves.
16239	Anano	Convolvulaceae	*Convolvulus *sp.	Eaten by cattle and mountain nyala.
16349	No name	Convolvulaceae	*Dichondra repens *J.R. Forst. & G. Forst.	Construction; used to build fences because of the spines.
16311	Anamo	Convolvulaceae	*Ipomoea *sp.	Eaten by cattle and mountain nyala.
16410	No name	Convolvulaceae	*Ipomoea *sp.	No use.
16173	No name	Crassulaceae	*Crassula alba *Forssk.	Eaten by baboons (root).
16081	No name	Crassulaceae	*Crassula *cf *schimperi *Fisch. & A. Mey.	Eaten by baboons. NOTE: "This grows on rocks."
16086	An'chura	Crassulaceae	*Kalanchoe petitiana *A. Rich.	Medicine; heat the leaves over the fire and apply to relax sore muscles.
16086	An'chura	Crassulaceae	*Kalanchoe petitiana *A. Rich.	Veterinary; if cattle break their legs, warm the plant or leaves over the fire and rub on the broken bone.
16068	No name	Crassulaceae	*Sedum baleensis *M. Gilbert	No use.
16090	Angudula	Crassulaceae	*Sedum *cf *churchillianum *Robyns & Boutique	Poison; in former times used to poison Hyenas. The plant material is crushed and out in meat. This makes the Hyena weak and intoxicated so that it can be killed.
15996	No name	Crassulaceae	*Umbilicus botryoides *A. Rich.	No use. NOTE: "It grows on trees."
16444	No name	Crassulaceae		No use.
16359	Han'chote	Cucurbitaceae	*Cucumis ficifolius *A. Rich.	Medicine; fruits are warmed in the fire and then smeared on infected wound etc. This will extract the pus.
16359	Han'chote	Cucurbitaceae	*Cucumis ficifolius *A. Rich.	Medicine; the roots are crushed and drunk with coffee for sudden illness, e.g. when someone colapses or has sudden stomach problems.
16117	Alola	Cucurbitaceae	*Zehneria scabra *(L.f.) Sond.	Eaten (fed especially to calves).
16117	Alola	Cucurbitaceae	*Zehneria scabra *(L.f.) Sond.	To make ink. In former times crushed and mixed with charcoal.
16335	Haloa	Cucurbitaceae		Eaten by cattle.
16424	Hindesa	Cupressaceae	*Juniperus procera *Hochst. ex Endl.	Construction.
16424	Hindesa	Cupressaceae	*Juniperus procera *Hochst. ex Endl.	Firewood.
16250	No name	Cuscutaceae	*Cuscuta kilimanjari *Oliv.	Eaten by cattle and mountain nyala. NOTE: Recognized as parasite.
16250	No name	Cuscutaceae	*Cuscuta kilimanjari *Oliv.	Flowers for honey. NOTE: Recognized as parasite.
16219	D'jafa	Cyperaceae	*Carex *cf *simensis *Hochst. ex A. Rich.	Eaten by cattle.
16219	D'jafa	Cyperaceae	*Carex *cf *simensis *Hochst. ex A. Rich.	Thatch.
16006	Mata	Cyperaceae	*Carex johnstonii *Boeck.	Eaten by animals.
16006	Mata	Cyperaceae	*Carex johnstonii *Boeck.	Thatch.
16084	Ch'afa	Cyperaceae	*Cyperus *cf *bracheilema *(Steud.) Mattf. & Kük.	Eaten by cattle.
16218	Arbagadda	Cyperaceae	*Cyperus dichroostachyus *Hochst. ex A. Rich.	Eaten by cattle.
16218	Arbagadda	Cyperaceae	*Cyperus dichroostachyus *Hochst. ex A. Rich.	Weave floor mats.
16345	Bidara	Cyperaceae	*Cyperus *sp.	Eaten by cattle.
16085	No name	Cyperaceae	*Isolepis *cf *omissa *J. raynal	Eaten by baboons.
16023	Gwemagni/Talandu	Cyperaceae	*Isolepis setacea *(L.) R. Br.	Eaten by cattle.
16023	Gwemagni/Talandu	Cyperaceae	*Isolepis setacea *(L.) R. Br.	Thatch.
16292	Aneno	Dioscoreaceae	*Dioscorea *sp.	Eaten by cattle and mountain nyala. Cattle really like this.
16179	No name	Dipsacaceae	*Dipsacus pinnatifidus *Steud. ex A. Rich.	No use.
16123	No name	Dipsacaceae	*Scabiosa columbaria *L.	No use.
16024	No name	Dracaenaceae	*Dracaena afromontana *Mildbr.	No use.
16412	Kokosa	Dryopteridaceae	*Doryopteris concolor *(Langsd. & Fisch.) Kuhn	No use.
16016	Kokosa	Dryopteridaceae	*Dryopteris *sp.	Sometimes eaten by cattle and mountain nyala.
16100	Kokosa	Dryopteridaceae	*Dryopteris *sp.	No use.
16199	Kokosa	Dryopteridaceae	*Dryopteris *sp.	No use.
16269	Kokosa	Dryopteridaceae	*Dryopteris *sp.	No use.
16019	Kokosa	Dryopteridaceae	*Polystichum fuscopaleaceum *Alston	No use. NOTE: "At high altitude it is red, at low altitude white."
16270	Kokosa	Dryopteridaceae		No use.
16096	Sato	Ericaceae	*Erica arborea *L.	Eaten by cattle.
16096	Sato	Ericaceae	*Erica arborea *L.	Firewood.
16096	Sato	Ericaceae	*Erica arborea *L.	Flowers for honey.
16194	Tula	Ericaceae	*Myrsine melanophloeos *(L.) R. Br.	Construction (Timber).
16194	Tula	Ericaceae	*Myrsine melanophloeos *(L.) R. Br.	Firewood.
16221	No name	Euphorbiaceae	*Acalypha volkensii *Pax	No use.
16138	F'eo	Euphorbiaceae	*Clutia abyssinica *Jaub. & Spach.	No use.
16251	Makanisa	Euphorbiaceae	*Croton macrostachyus *Hochst. ex Delile	Medicine; sometimes the bark is mixed with Hanku to produce better stomach medicine.
16188	Guri	Euphorbiaceae	*Euphorbia dumalis *S. Carter	Medicine; crush a little of the root, mix with coffee and honey and drink against Syphilis.
16188	Guri	Euphorbiaceae	*Euphorbia dumalis *S. Carter	NOT eaten by cattle.
16116	Guri	Euphorbiaceae	*Euphorbia schimperiana *Scheele	Medicine; crush a little of the root, mix with coffee and honey and drink against Syphilis.
16378	No name	Euphorbiaceae	*Euphorbia *sp.	No use.
15992	No name	Euphorbiaceae	*Phyllanthus *cf *ovalifolius *Forssk.	No use.
16312	No name	Euphorbiaceae	*Phyllanthus *cf *ovalifolius *Forssk.	No use.
16416	Kobo	Euphorbiaceae	*Ricinus communis *L.	Eaten by cattle.
16416	Kobo	Euphorbiaceae	*Ricinus communis *L.	Food; seeds are crushed and the oil extracted to grease the pans for baking bread.
16416	Kobo	Euphorbiaceae	*Ricinus communis *L.	To soften leather. Fruits crushed and applied to leather to soften.
16369	Dadetsha	Fabaceae	*Acacia abyssinica *Hochst. ex Benth.	Charcoal (preferred species).
16369	Dadetsha	Fabaceae	*Acacia abyssinica *Hochst. ex Benth.	Eaten by camels and goats.
16369	Dadetsha	Fabaceae	*Acacia abyssinica *Hochst. ex Benth.	Firewood.
16347	Tshe'kata	Fabaceae	*Caesalpinia *sp.	Tools; wood used to make ploughs.
16413	Chekata	Fabaceae	*Caesalpinia *sp.	Firewood.
16035	Shashamane	Fabaceae	*Crotalaria rosenii *(Pax) Milne-Redh. ex Polhill	Eaten by goats and sheep. They really like this.
15989	No name	Fabaceae	*Desmodium repandum *(Vahl) DC.	No use.
16232	Walena	Fabaceae	*Erythrina brucei *Schweinf.	Food; leaves used to wrap dough when making bread.
16232	Walena	Fabaceae	*Erythrina brucei *Schweinf.	Tools; wood used to make the pestle for large mortars.
15991	Sedisa	Fabaceae	*Medicago *sp.	Eaten by cattle.
16149	Kumudo	Fabaceae	*Medicago *sp.	Eaten by cattle. NOTE: This name applied to samples without fruits.
16343	Dadatu	Fabaceae	*Millettia ferruginea *(Hochst.) Baker	Construction (timber).
16343	Dadatu	Fabaceae	*Millettia ferruginea *(Hochst.) Baker	Firewood.
16034	Sedisa	Fabaceae	*Parochetus communis *Buch.-Ham. ex D. Don.	Eaten by cattle.
16387	Shiko	Fabaceae	*Senna *sp.	No use.
15994	Sedisa	Fabaceae	*Trifolium semipilosum *Fresen.	Eaten by cattle.
16060	No name	Fabaceae	*Trifolium simense *Fresen.	Eaten by baboons.
16157	No name	Fabaceae	*Trifolium *sp.	Eaten by cattle.
16242	Anano	Fabaceae		Eaten by livestock and wildlife.
16284	No name	Fabaceae		Eaten by cattle.
16285	Anjakere	Fabaceae		Eaten by cattle.
16348	Gorsana	Fabaceae		Construction; used to build fences because of the spines.
16364	No name	Fabaceae		No use.
16385	Sidika	Fabaceae		No use.
16411	No name	Fabaceae		No use.
16439	Anjakere	Fabaceae		Making brooms.
16406	Dembi	Flacourtiaceae	*Casearia *sp.	Firewood.
16208	Koshimo/Koshima	Flacourtiaceae	*Dovyalis abyssinica *(A. Rich.) Warb.	Eaten by cattle.
16208	Koshimo/Koshima	Flacourtiaceae	*Dovyalis abyssinica *(A. Rich.) Warb.	Firewood.
16208	Koshimo/Koshima	Flacourtiaceae	*Dovyalis abyssinica *(A. Rich.) Warb.	Food; fruits eaten, but they are very sour.
16321	Riga	Flacourtiaceae		Toothbrush.
16346	Diki	Flacourtiaceae		Eaten by cattle and mountain nyala.
16346	Diki	Flacourtiaceae		Rope; used to hang beehives.
16399	No name	Flacourtiaceae		No use.
16074	No name	Gentianaceae	*Sebaea brachyphylla *Griseb.	No use.
16062	No name	Gentianaceae	*Swertia *sp.	Eaten by baboons.
16126	No name	Gentianaceae	*Swertia *sp.	No use.
16170	No name	Gentianaceae	*Swertia *sp.	No use.
16109	No name	Geraniaceae	*Geranium aculeolatum *Oliv.	Eaten by cattle and mountain nyala.
16080	No name	Geraniaceae	*Geranium arabicum *Forssk.	Eaten by cattle.
16373	No name	Geraniaceae	*Geranium arabicum *Forssk.	No use.
16438	No name	Iridaceae		No use.
16102	Ch'afa	Juncaceae	*Luzula johnstonii *Buchenau	Eaten by cattle when the plant is very young.
16102	Ch'afa	Juncaceae	*Luzula johnstonii *Buchenau	Thatch.
16295	Sukaiahareti	Lamiaceae	*Achyrospermum schimperi *(Briq.) Perkins	No use.
16160	Tosin	Lamiaceae	*Becium *cf *obovatum *(E. Mey. ex Benth.) N.E. Br.	Food; used to make tea, as spice for butter, and as spice for baso (roasted and ground barley).
16310	Burasisa	Lamiaceae	*Clerodendrum myricoides *(Hochst.) R. Br. ex Vatke	Eaten by cattle.
16137	Informant does not remember name	Lamiaceae	*Leonotis nepetifolia *(L.) R. Br.	Food; children like to suck the nectar from the flowers.
16137	Informant does not remember name	Lamiaceae	*Leonotis nepetifolia *(L.) R. Br.	Medicine; crush the plant and take as tea for stomach ailments.
16225	Urgo	Lamiaceae	*Leucas martinicensis *(Jacq.) R. Br.	Eaten by cattle.
16316	Korichi Michi/Damakase	Lamiaceae	*Ocimum *sp.	Medicine; for intestinal infections like cold or flu. Crush the leaves, add a little water, inhale juice or smear on body or drunk with coffee.
16332	Urgohare	Lamiaceae	*Ocimum *sp.	No use.
16128	Tunto	Lamiaceae	*Otostegia tomentosa *A. Rich.	Birds get nectar.
16128	Tunto	Lamiaceae	*Otostegia tomentosa *A. Rich.	NOT eaten by cattle.
16079	Burri	Lamiaceae	*Plectranthus *sp.	Eaten by cattle and baboons.
16087	No name	Lamiaceae	*Plectranthus *sp.	No use.
16097	Burri	Lamiaceae	*Plectranthus *sp.	Eaten by cattle.
16286	Damakasi	Lamiaceae	*Plectranthus *sp.	Eaten by cattle.
16286	Damakasi	Lamiaceae	*Plectranthus *sp.	Medicine; leaves are crushed and the extract drunk for colds, or leaves are crushed and stuck in the nose to treat colds.
16287	Urgo	Lamiaceae	*Plectranthus *sp.	Eaten by cattle.
16287	Urgo	Lamiaceae	*Plectranthus *sp.	Medicine; leaves crushed and put on wounds.
16356	Ocota	Lamiaceae	*Salvia merjamie *Forssk.	Eaten by cattle.
16356	Ocota	Lamiaceae	*Salvia merjamie *Forssk.	Medicine; leaves crushed and smeared on the body to treat any unknown or undiagnosed illness.
16001	Ocota	Lamiaceae	*Salvia nilotica *Juss. ex Jacq.	Eaten by cattle and mountain nyala.
16098	No name	Lamiaceae	*Satureja *sp.	No use.
16177	No name	Lamiaceae	*Satureja *sp.	No use.
15997	Informant does not remember name	Lamiaceae	*Stachys aculeolata *Hook. f.	Eaten by cattle and mountain nyala.
16178	No name	Lamiaceae	*Stachys *sp.	No use.
16163	No name	Lamiaceae	*Thymus schimperi *Ronniger	Eaten by cattle.
15995	Burri	Lamiaceae		Flowers for honey.
15995	Burri	Lamiaceae		NOT eaten by anything.
16389	Abaye	Lauraceae	*Ocotea kenyensis *(Chiov.) Robyns & R. Wilczek	Firewood.
16391	Apeyu	Lauraceae		Construction (house).
16391	Apeyu	Lauraceae		Firewood.
16066	No name	Liliaceae		Eaten by baboons who really like it, especially the roots. Fruit smells like garlic.
16215	No name	Loranthaceae	*Englerina woodfordioides *(Schweinf.) Balle ex M.G. Gilbert	No use.
16187	No name	Lycopodiaceae	*Huperzia dacrydioides *(Baker) Pic. Serm.	No use.
16268	Danisa	Malvaceae	*Dombeya kirkii *Mast.	Eaten by cattle.
16268	Danisa	Malvaceae	*Dombeya kirkii *Mast.	Flowers for honey.
16268	Danisa	Malvaceae	*Dombeya kirkii *Mast.	Rope. Bark peeled for rope used in construction.
16323	Danisa	Malvaceae	*Dombeya *sp.	Eaten by cattle.
16323	Danisa	Malvaceae	*Dombeya *sp.	Firewood.
16323	Danisa	Malvaceae	*Dombeya *sp.	Flowers for honey.
16058	Danisa	Malvaceae	*Dombeya torrida *Bamps.	Eaten by cattle.
16118	Danisa	Malvaceae	*Dombeya torrida *Bamps.	Flowers for honey.
16331	Matakoma	Malvaceae	*Grewia *sp.	Firewood.
16267	Ishini	Malvaceae	*Hibiscus *sp.	Eaten by cattle and mountain nyala.
16267	Ishini	Malvaceae	*Hibiscus *sp.	Rope; bark peeled for rope used in construction.
16318	No name	Malvaceae	*Hibiscus *sp.	No use.
16003	Amoja/Amocha	Malvaceae	*Kosteletzkya adoensis *(Hochst. ex A. Rich.) Mast.	Eaten by cattle and mountain nyala.
16003	Amoja/Amocha	Malvaceae	*Kosteletzkya adoensis *(Hochst. ex A. Rich.) Mast.	Toothbrush.
16130	Lita	Malvaceae	*Malva *sp.	Eaten by cattle.
16130	Lita	Malvaceae	*Malva *sp.	Medicine; in the past used to stimulate hair growth. Plant crushed and applied to the hair.
16317	Ishini	Malvaceae	*Pavonia *sp.	Eaten by cattle and mountain nyala.
16317	Ishini	Malvaceae	*Pavonia *sp.	Rope. Bark peeled and used as rope for construction.
16067	Hamoja	Malvaceae	*Sparmannia ricinocarpa *(Eckl. & Zeyh.) Kuntze	Eaten by Giant forest hog.
16224	Amoja	Malvaceae	*Triumfetta rhomboidea *Jacq.	Eaten by mountain nyala, but NOT eaten by cattle.
16211	No name	Malvaceae		No use.
16245	Ishini	Malvaceae		Flowers for honey.
16245	Ishini	Malvaceae		Rope; bark peeled for rope used in construction.
16298	Injin	Malvaceae		Eaten by cattle and mountain nyala.
16298	Injin	Malvaceae		Rope. Bark peeled and used as rope for construction.
16324	Anunu	Meliaceae	*Ekebergia capensis *Sparm.	Firewood.
16234	Harambe	Meliaceae	*Lepidotrichilia volkensii *(Gürke) J.-F. Leroy	Firewood.
16234	Harambe	Meliaceae	*Lepidotrichilia volkensii *(Gürke) J.-F. Leroy	Tools; wood to make ploughs.
16259	Hacho	Meliaceae	*Lepidotrichilia volkensii *(Gürke) J.-F. Leroy	Firewood.
16390	Hacho	Meliaceae	*Trichilia dregeana *Sond.	Firewood.
16309	Bulala	Meliaceae	*Turraea holstii *Gürke	No use.
16029	Arambye	Melianthaceae	*Bersama abyssinica *Fresen.	Tools; wood to make ploughs.
16342	Oroka	Melianthaceae	*Bersama abyssinica *Fresen.	Firewood.
16183	Kalala	Menispermaceae	*Stephania abyssinica *(Quart.-Dill. & A. Rich.) Walp.	Rope; to tie houseposts.
16183	Kalala	Menispermaceae	*Stephania abyssinica *(Quart.-Dill. & A. Rich.) Walp.	Tools; to make containers for miliking.
16203	No name	Moraceae	*Dorstenia soerensenii *Friis	No use.
16341	Dembi	Moraceae	*Ficus *sp.	Firewood.
16403	Lint'o	Moraceae	*Ficus *sp.	Firewood.
16340	Oda	Moraceae	*Ficus sur *Forssk.	Beehives (wood).
16340	Oda	Moraceae	*Ficus sur *Forssk.	Food; fruits eaten by humans.
16340	Oda	Moraceae	*Ficus sur *Forssk.	NOT eaten by cattle
16402	Oda	Moraceae	*Ficus sycomorus *L.	Beehives (wood).
16402	Oda	Moraceae	*Ficus sycomorus *L.	Food; fruits eaten by monkeys, baboons and humans.
16306	No name	Moraceae		No use.
16028	Hanku	Myrsinaceae	*Embelia schimperi *Vatke	Eaten by goats, sheep, cattle, mountain nyala and baboons.
16028	Hanku	Myrsinaceae	*Embelia schimperi *Vatke	Medicine; the fruits and stems are used as anthelmintic. Eat what fits in the bowl of a hand.
16260	Hanku	Myrsinaceae	*Embelia schimperi *Vatke	Eaten by cattle and mountain nyala.
16260	Hanku	Myrsinaceae	*Embelia schimperi *Vatke	Medicine; crush fruits and drink liquid to cure tapeworm.
16022	Abeye	Myrsinaceae	*Myrsine africana *L.	Food; women use the leaves to roll the dough in before putting it in the oven so that it does not buirn. The seeds are crused and the oil is used to grease the baking plate before baking.
16272	Badesa	Myrtaceae	*Syzygium guineense *(Willd.) DC.	Firewood.
16272	Badesa	Myrtaceae	*Syzygium guineense *(Willd.) DC.	Flowers for honey. NOTE: Main honey source.
16272	Badesa	Myrtaceae	*Syzygium guineense *(Willd.) DC.	Food; fruits eaten by humans.
16278	Badesa	Myrtaceae	*Syzygium guineense *(Willd.) DC.	Firewood.
16278	Badesa	Myrtaceae	*Syzygium guineense *(Willd.) DC.	Flowers for honey. NOTE: Main honey source.
16278	Badesa	Myrtaceae	*Syzygium guineense *(Willd.) DC.	Food; fruits eaten by humans.
16405	Badesa	Myrtaceae	*Syzygium guineense *(Willd.) DC.	Construction (house).
16405	Badesa	Myrtaceae	*Syzygium guineense *(Willd.) DC.	Firewood.
16405	Badesa	Myrtaceae	*Syzygium guineense *(Willd.) DC.	Food; fruit eaten by humans.
16374	No name	Nyctaginaceae	*Boerhavia *sp.	No use.
16375	No name	Nyctaginaceae	*Boerhavia *sp.	No use.
16112	T'orso	Oleaceae	*Jasminum abyssinicum *Hochst. ex. DC.	Eaten (especially fed to calves).
16112	T'orso	Oleaceae	*Jasminum abyssinicum *Hochst. ex. DC.	Eaten by cattle.
16112	T'orso	Oleaceae	*Jasminum abyssinicum *Hochst. ex. DC.	Rope; used in house construction.
16112	T'orso	Oleaceae	*Jasminum abyssinicum *Hochst. ex. DC.	Toothbrush (young stems).
16329	Badesa	Oleaceae	*Olea hochstetteri *Baker	Construction (timber).
16329	Badesa	Oleaceae	*Olea hochstetteri *Baker	Food; fruit eaten by humans.
16329	Badesa	Oleaceae	*Olea hochstetteri *Baker	NOT eaten by cattle.
16124	No name	Orobanchaceae	*Orobanche minor *Sm.	No use.
16106	No name	Oxalidaceae	*Oxalis *sp.	Eaten by cattle.
16386	Korehare	Papaveraceae	*Argemone mexicana *L.	Eaten by donkeys. NOTE: Name translates to "spiny donkey".
16121	Endode	Phytolaccaceae	*Phytolacca dodecandra *L.Hér.	Detergent; fruits crushed and used to wash cloths.
16121	Endode	Phytolaccaceae	*Phytolacca dodecandra *L.Hér.	Medicine; roots chewed for stomach problems. The fruits kill water animals and can be used to prevent Bilharzia.
16121	Endode	Phytolaccaceae	*Phytolacca dodecandra *L.Hér.	Toothbrush (twigs).
16301	No name	Phytolaccaceae		Eaten by cattle.
16008	Kontuyesa	Piperaceae	*Peperomia abyssinica *Miq.	Medicine; boil the leaves and apply the steam to pimples and abscesses.
16314	No name	Piperaceae	*Peperomia tetraphylla *(G. Forst.) Hook. & Arn.	No use.
16193	Ara	Pittosporaceae	*Pittosporum abyssinicum *Delile	Eaten by cattle and mountain nyala.
16193	Ara	Pittosporaceae	*Pittosporum abyssinicum *Delile	Firewood.
16193	Ara	Pittosporaceae	*Pittosporum abyssinicum *Delile	Toothbrush (small branches).
16352	Name unknown	Plantaginaceae	*Plantago lanceolata *L.	Eaten by cattle.
16428	No name	Plantaginaceae	*Plantago palmata *Hook. f.	No use.
16005	Garaba	Poaceae	*Acritochaete volkensii *Pilg.	Eaten by livestock and wildlife.
16434	Tuta	Poaceae	*Avenula *sp.	Eaten by cattle.
16050	Garaba	Poaceae	*Bromus leptoclados *Nees	Eaten by cattle.
16050	Garaba	Poaceae	*Bromus leptoclados *Nees	Thatch.
16103	Mata	Poaceae	*Calamagrostis epigejos *(L.) Roth.	NOT eaten by cattle.
16103	Mata	Poaceae	*Calamagrostis epigejos *(L.) Roth.	Thatch.
16249	Korcha	Poaceae	*Digitaria *sp.	Eaten by cattle and mountain nyala.
16253	Hat'aua	Poaceae	*Elymus *sp.	Eaten by cattle.
16253	Hat'aua	Poaceae	*Elymus *sp.	Thatch.
16082	Marga	Poaceae	*Eragrostis cilianensis *(Bellardi) Vignolo ex. Janch.	Eaten by cattle. NOTE: Marga simply translates to grass.
16264	Garaba	Poaceae	*Oplismenus compositus *(L.) P. Beauv.	Eaten by cattle and mountain nyala.
16299	Marga	Poaceae	*Panicum *sp.	Eaten by cattle and mountain nyala.
16248	Chokorsa	Poaceae	*Paspalum *sp.	Eaten by cattle and mountain nyala.
16290	Babala/Babela	Poaceae	*Paspalum *sp.	Eaten by cattle and mountain nyala.
16351	Marga	Poaceae	*Poa annua *L.	Eaten by cattle. NOTE: This plant had to be examined very closely before consent could be reached.
16004	Marga	Poaceae	*Poa leptoclada *A. Rich.	Eaten by cattle.
16262	Babala/Babela	Poaceae	*Stipa dregeana *Steudl.	Eaten by cattle.
16049	Garaba	Poaceae	*Streblochaete longiarista *(A. Rich.) Pilg.	Eaten by cattle.
16049	Garaba	Poaceae	*Streblochaete longiarista *(A. Rich.) Pilg.	Thatch.
16265	Garaba	Poaceae	*Streblochaete longiarista *(A. Rich.) Pilg.	Eaten by cattle and mountain nyala.
16083	Marga	Poaceae		Eaten by cattle. NOTE: Marga simply translates to grass.
16252	Mata	Poaceae		Eaten by cattle and mountain nyala.
16252	Mata	Poaceae		Thatch.
16291	Babala/Babela	Poaceae		Eaten by cattle and mountain nyala.
16300	Marga	Poaceae		Eaten by cattle and mountain nyala.
16433	Mata	Poaceae		Eaten by cattle.
16433	Mata	Poaceae		Thatch.
16246	Bosoka	Polygonaceae	*Polygonum afromontanum *Greenway	No use.
16227	Berberisa	Polygonaceae	*Rumex abyssinicus *Jacq.	No use.
16009	Shabee	Polygonaceae	*Rumex nepalensis *Spreng.	Eaten by cattle.
16009	Shabee	Polygonaceae	*Rumex nepalensis *Spreng.	Medicine; roots are crushed and eaten for stomach problems.
16009	Shabee	Polygonaceae	*Rumex nepalensis *Spreng.	Veterinary; leaves are crushed and given to livestock for stomach problems.
16360	Haberira	Polygonaceae	*Rumex *sp.	Medicine; root is crushed, and water added and drunk when somebody "feels that something is worng," i.e. has an undiagnosed illness.
16189	Kokosa	Polypodiaceae	*Drynaria volkensii *Hieron.	No use.
16018	No name	Polypodiaceae	*Loxogramme abyssinica *(Baker) M.G. Price	No use. NOTE: "At high altitude it is red, at low altitude white."
16392	No name	Portulacaceae	*Portulaca oleracea *L.	No use.
16070	No name	Primulaceae	*Ardisiandra wettsteinii *R. Wagner	Eaten by mountain nyala.
16154	Matane	Primulaceaea	*Anagallis arvensis *L.	Eaten by cattle. NOTE: "There are two kinds in the area."
16154	Matane	Primulaceaea	*Anagallis arvensis *L.	Medicine. Applied to the eye to improve "fat yellow thing" in the eye. NOTE: "There are two kinds in the area."
16119	No name	Pteridaceae	*Adiantum capillus-veneris *L.	No use.
16101	Kokosa	Pteridaceae	*Cheilanthes farinosa *(Forssk.) Kaulf.	No use.
16200	Kokosa	Pteridaceae	*Cheilanthes farinosa *(Forssk.) Kaulf.	No use.
16217	No name	Pteridaceae	*Pteridium aquilinum *(L.) Kuhn	No use.
16015	Kokosa	Pteridaceae	*Pteris catoptera *Kunze	Sometimes eaten by cattle and mountain nyala.
16271	Kokosa	Pteridaceae	*Pteris catoptera *Kunze	No use.
16261	Kokosa	Pteridaceae	*Pteris dentata *Forssk.	No use.
16201	Kokosa	Pteridaceae	*Pteris flabellata *Thunb.	No use.
16420	Kokosa	Pteridaceae	*Pteris *sp.	No use.
16396	Kokosa	Pteridaceae		No use.
16047	Fidy	Ranunculaceae	*Clematis bracteata *(Roxb.) Kurz	Medicine; used for "lung cancer" and cancer in general. The plant is crushed and mixed with butter and applied to wounds, haemorrhoids and burns.
16282	Hacho	Ranunculaceae	*Clematis hirsuta *Guill. & Perr.	Eaten by cattle and mountain nyala.
16304	Fidy	Ranunculaceae	*Clematis simensis *Fresen.	Rope; bark peeled and used as rope for construction.
16059	No name	Ranunculaceae	*Delphinium wellbyi *Hemsl.	No use.
16427	No name	Resedaceae	*Caylusea abyssinica *Fisch. & C.A. Mey.	No use.
16325	No name	Rhamnaceae	*Helinus integrifolius *(Lam.) Kuntze	No use.
16431	Gesho	Rhamnaceae	*Rhamnus prinoides *L'Hér.	Food; leaves used to flavor beer and mead. Only used by Christians. Very bitter.
16365	Bitana	Rhamnaceae	*Rhamnus staddo *A. Rich.	Construction (house).
16045	Thelo	Rhizophoraceae	*Cassipourea malosana *(Baker) Alston	Firewood.
16045	Thelo	Rhizophoraceae	*Cassipourea malosana *(Baker) Alston	Tools; wood used to make ploughs.
16169	Sedisa	Rosaceae	*Alchemilla fischeri *Engl.	Eaten by cattle and baboons.
16181	Sedisa	Rosaceae	*Alchemilla kiwuensis *Engl.	Eaten by cattle.
16057	Heto	Rosaceae	*Hagenia abyssinica *J.F. Gmel.	Eaten by cattle.
16057	Heto	Rosaceae	*Hagenia abyssinica *J.F. Gmel.	Medicine; fruits are dried, ground and eaten before a meal as anthelmintic. Better taken early in the morning with some food.
16226	Suke	Rosaceae	*Prunus africana *(Hook. f.) Kalkman	Rope; used to hang beehives.
16192	Gora	Rosaceae	*Rubus steudneri *Schweinf.	Eaten especially by mountain nyala.
16192	Gora	Rosaceae	*Rubus steudneri *Schweinf.	Food; Fruit eaten by people.
16030	Coralla	Rubiaceae	*Canthium oligocarpum *Hiern	Eaten by cattle and mountain nyala.
16030	Coralla	Rubiaceae	*Canthium oligocarpum *Hiern	Food; fruits eaten by children.
16275	Coralla	Rubiaceae	*Canthium *sp.	Firewood.
16275	Coralla	Rubiaceae	*Canthium *sp.	Food; fruits eaten by humans.
16276	Gagama	Rubiaceae	*Canthium *sp.	Tools; wood used to make ploughs.
16305	Buna	Rubiaceae	*Coffea arabica *L.	Food; fallen leaves roasted for tea, seeds coffee.
16180	Matane	Rubiaceae	*Galium aparinoides *Forssk.	Eaten by cattle.
16164	No name	Rubiaceae	*Oldenlandia monanthos *(Hochst. ex A. Rich.) Hiern	Eaten by cattle.
16051	No name	Rubiaceae	*Pentas schimperiana *(A. Rich.) Vatke	Eaten by cattle.
16283	No name	Rubiaceae	*Pentas *sp.	Eaten by cattle and other animals.
16415	Bulala	Rubiaceae	*Psydrax *sp.	Firewood.
16258	Farangasa	Rubiaceae	*Rhytigynia *sp.	Firewood.
16426	No name	Rubiaceae	*Rubia cordifolia *L.	Medicine for "snake spit that causes wounds." Root and leaves are chewed and then spit on the affected area.
16255	Sugurgura	Rubiaceae	*Vangueria *sp.	Construction; to link the main poles and the roof beams.
16307	Arbo	Rutaceae	*Citrus aurantium *L.	Food; fruits eaten by baboons and humans.
16233	H'adesa	Rutaceae	*Teclea nobilis *Delile	NOT used for firewood.
16233	H'adesa	Rutaceae	*Teclea nobilis *Delile	Tools; wood to make ploughs.
16257	Adesa	Rutaceae	*Teclea *sp.	Firewood.
16274	Adesa	Rutaceae	*Teclea *sp.	Firewood.
16315	Harira	Rutaceae	*Teclea *sp.	Firewood.
16417	Sadiqua	Rutaceae	*Toddalia asiatica *(L.) Lam.	No use.
16273	Harera	Rutaceae	*Vepris dainellii *(Pic. Seerm.) Miziray	Firewood.
16401	Gagama	Rutaceae		Tools; wood used to make ploughs.
16436	Bitana	Rutaceae		Firewood.
16339	Deerto	Santalaceae	*Viscum triflorum *DC.	No use.
16044	Habarra	Sapindaceae	*Allophylus abyssinicus *(Hochst.) Radkl.	Food; Fruits eaten by children.
16044	Habarra	Sapindaceae	*Allophylus abyssinicus *(Hochst.) Radkl.	Medicine; Flowers for Honey; This honey has medicinal properties and is good for stomach problems.
16277	Aberra	Sapindaceae	*Allophylus abyssinicus *(Hochst.) Radkl.	Eaten by cattle.
16277	Aberra	Sapindaceae	*Allophylus abyssinicus *(Hochst.) Radkl.	Food; Fruits eaten by humans and baboons.
16256	Guduba	Sapotaceae	*Aningeria adolfi-friederici *(Engl.) Robyns & G.C.C. Gilbert	Firewood.
16072	No name	Saxifragaceae	*Saxifraga *sp.	Eaten by baboons.
16075	No name	Saxifragaceae	*Saxifraga *sp.	No use.
16167	No name	Scrophulariaceae	*Bartsia petitiana *(A. Rich.) Hemsl.	No use.
16437	No name	Scrophulariaceae	*Bartsia *sp.	No use.
16092	Bulchana	Scrophulariaceae	*Buddleja polystachya *Fresen.	Firewood.
16158	Kankarasho	Scrophulariaceae	*Craterostigma pumilum *Hochst.	Eaten by baboons (root).
16158	Kankarasho	Scrophulariaceae	*Craterostigma pumilum *Hochst.	Food; Roots chewed by humans for taste (like a sweet carrot)
16071	No name	Scrophulariaceae	*Craterostigma *sp.	Eaten by baboons.
16156	Bilike	Scrophulariaceae	*Cycniopsis humilis *A. Bacjklund, Asfaw & Långström	Eaten by baboons (root).
16159	No name	Scrophulariaceae	*Hebenstretia angolensis *Rolfe	No use.
16064	No name	Scrophulariaceae	*Rhabdotosperma *sp.	No use.
16429	Ashishira	Scrophulariaceae	*Verbascum sinaiticum *Benth.	Making brooms.
16069	No name	Scrophulariaceae	*Veronica abyssinica *Fresen.	No use.
16107	Da'ta	Scrophulariaceae	*Veronica glandulosa *Hochst. ex Benth.	Eaten by cattle.
16443	Hada	Scrophulariaceae		Eaten by cattle.
16443	Hada	Scrophulariaceae		Flowers for honey.
16445	No name	Scrophulariaceae		No use.
16140	Ironto	Simaroubaceae	*Brucea antidysenterica *J.F. Mill.	NOT eaten by animals, not even by ants. The smoke and the plant smell bad. Can be used as insect repellent.
16363	Banji	Solanaceae	*Datura stramonium *L.	Medicine; Leaves are crushed and applied to abscesses with pus, deep thorns, infected swollen wounds etc. This will extract the thorn/pus. NOTE: The name translates to "plant from the east" in Amharic: Atafaris. It is also used as generic name for "drug".
16363	Banji	Solanaceae	*Datura stramonium *L.	Poison. Seeds might acidentially get mixed with barley. If that is eaten one gets intoxicated, the mouth gets dry, and the mind gets confused. The effect starts as soon as the digestion has happened.
16042	Marraro	Solanaceae	*Discopodium penninervium *Hochst.	Eaten by horses.
16381	Benjisaria	Solanaceae	*Physalis *sp.	Weed.
16319	Hiddi	Solanaceae	*Solanum incanum *L.	Eaten by cattle and goats.
16319	Hiddi	Solanaceae	*Solanum incanum *L.	Medicine; roots chewed for stomach health and sudden pain.
16319	Hiddi	Solanaceae	*Solanum incanum *L.	Poison; fruits are toxic.
16370	Hiddi	Solanaceae	*Solanum incanum *L.	Eaten by cattle (leaves).
16370	Hiddi	Solanaceae	*Solanum incanum *L.	Medicine; root is eaten for stomach problems of sudden onset. Very bitter.
16231	Hiddi	Solanaceae	*Solanum macracanthum *A. Rich.	Medicine; root chewed for stomach problems.
16231	Hiddi	Solanaceae	*Solanum macracanthum *A. Rich.	NOT eaten by cattle.
16362	Mijilo	Solanaceae	*Solanum nigrum *L.	Eaten by cattle.
16362	Mijilo	Solanaceae	*Solanum nigrum *L.	Food; children eat the fruits.
16371	Mishilo Huarabesa	Solanaceae	*Solanum *sp.	No use. NOTE: Name translates as "Hyena tomato".
16372	Hiti'arbo	Solanaceae	*Solanum *sp.	Detergent; fruits were formerly boiled and used as detergent to wash cloths.
16129	Unso	Solanaceae	*Withania somnifera *(L.) Dunal	Medicine; roots are smashed or chewed like a carrot to treat stomach problems. The leaves are burnt as incense as spiritual medicine.
16129	Unso	Solanaceae	*Withania somnifera *(L.) Dunal	NOT eaten by cattle.
16380	Hunso	Solanaceae		Veterinary. When the yoke hurts the back of the oxen and produces a swelling, crush the leaves and put on the swelling.
16118	Danisa	Sterculiaceae	*Dombeya torrida *Bamps.	Rope; bark peeled and used in construction. "They pull the water transport system for rope".
16367	Bitana	Stilbaceae	*Nuxia congesta *R. Br. ex Fresen.	Construction (house).
16367	Bitana	Stilbaceae	*Nuxia congesta *R. Br. ex Fresen.	Firewood.
16367	Bitana	Stilbaceae	*Nuxia congesta *R. Br. ex Fresen.	Flowers for honey.
16302	Kokosa	Tectariaceae	*Tectaria gemmifera *(Fée) Alston	No use.
16394	Kokosa	Thelypteridaceae	*Thelypteris *sp.	No use.
16395	Kokosa	Thelypteridaceae	*Thelypteris *sp.	No use.
16432	Didisa	Thymeleaceae	*Gnidia glauca *(Fresen.) Gilg	Firewood.
16432	Didisa	Thymeleaceae	*Gnidia glauca *(Fresen.) Gilg	Rope; bark used to make rope.
16184	No name	Urticaceae	*Pilea johnstonii *Oliv.	No use.
16185	No name	Urticaceae	*Pilea rivularis *Wedd.	Eaten by baboons.
16240	No name	Urticaceae	*Pilea *sp.	Eaten by cattle.
16056	Halila	Urticaceae	*Urera hypselodendron *Wedd.	Eaten by cattle and wildlife.
16254	Halila	Urticaceae	*Urera hypselodendron *Wedd.	No use.
16334	Lalesa	Urticaceae		No use.
16281	Sukai	Verbenaceae	*Lantana *sp.	Eaten by cattle.
16281	Sukai	Verbenaceae	*Lantana *sp.	Food; spice for butter, milk etc.
16425	No name	Verbenaceae	*Verbena *sp.	No use.
16280	No name	Verbenaceae		No use.
16327	Ulaga	Verbenaceae		Firewood.
16327	Ulaga	Verbenaceae		Tools; wood used to make ploughs.
16435	Dukunsha	Violaceae	*Viola abyssinica *Steud. ex Oliv.	To soften leather. Leaves are crushed to extract the juice, which is applied to hard leather.
16020	Lelecha	Vitaceae	*Cyphostemma *sp.	Poison; this burns your bowels when you eat it. NOT eaten by animals.
16418	No name	Vitaceae	*Rhoicissus *sp.	NOT eaten by cattle.
16063	No name	Xanthorrhoeaceae	*Asphodelus fistulosus *L.	Eaten by baboons.
16172	Lela	Xanthorrhoeaceae	*Kniphofia foliosa *Hochst.	No use.
16151	Ch'amare	Zygophyllaceae	*Tribulus terrestris *L.	Eaten by all animals.
16151	Ch'amare	Zygophyllaceae	*Tribulus terrestris *L.	Toothbrush (root).
16036	Seriti			Eaten sometimes by goats.
16149	Sedisa			Eaten by cattle. NOTE: This name applied to samples WITH fruits
16279	Wayebosa			Eaten by cattle.
16279	Wayebosa			Flowers for honey.
16421	Diki			Eaten by cattle.
16421	Diki			Rope.

A very limited number of species was used for veterinary purposes (13 species), or as human medicine (46 species). Many species however had multiple uses. Plant medicine served mostly to treat common everyday ailments such as stomach problems and diarrhea (9 species), for wound treatment and as toothbrush-sticks (6 species), as anthelmintic, for skin infections and to treat sore muscles and swellings (4 species each), or to foster hair growth, to treat colds, and syphilis (2 species each). One species was employed for female illnesses, and one to treat cancer. Interestingly, 9 species were used to treat spiritual ailments and to expel demons. In most cases of medicinal applications the leaves (26 species) or roots (15 species) were employed, while fruits (4 species), flowers (1 species) and bark (1 species) did not play a significant role.

Traditional plant knowledge has clearly declined in a large part of the research area. The most traditional groups still retain the highest knowledge of plant use for human purposes, although acculturated societies are shown to retain a much higher plant usage in order to treat common "modern" diseases such as sexually transmitted disease, as well as veterinary problems that are either stigmatized, for which western treatment does not prove effective, or for which cheap treatment cannot be found. Western style health care services as provided by governments and NGOs, in particular in rural areas, seem to have contributed to a decline in traditional knowledge, in part because the local population simply regards western medicine as more effective and safer, or as one of our Oromo informants put it *"Sick people go to the clinic or cultural practitioner who prepares medicine from plants. Nowadays people mostly go to the clinic. The head of household knows herbs and they might use these, but nowadays most people prefer to go to the clinic. Traditional herbalists are already very old. The tradition is normally passed from the father to the son. Formerly people came from far like from Addis, and there is still a woman healer who is famous for treatments for example for parasites. Western medicine is more scientific and thus more reliable. Traditional medicine is often very painful, and can cause harm. Sometimes people die of traditional medicine. For their animals people prefer to go to the animal hospital. Traditional remedies are only used for rabies."*

The knowledge of the Oromo population in both the highlands of Bale and the lower areas south of the massif were comparable. However, some profound differences were encountered. The Oromo of the Bale highlands did not use preparations for malaria for the simple fact that malaria does not exist in their area. Thirteen species were used as veterinary medicine by the Oromo in the study area. This is rather surprising, because [42] reported 74 veterinary medicinal plant species from the study region. Plants for the cure of venereal diseases such as Gonorrhea, Syphilis and others, were almost negligible in the present study in the Oromo area. Previous records indicate that venereal diseases were amongst the most frequently treated with plants amongst the Oromo [23,41].

These differences might indicate a clear difference in plant knowledge between traditional healers and laypeople. Experts clearly had a much more profound knowledge than the non-experts interviewed. We must also consider disparities in floral composition and availability between the Oromo people inhabiting our study area and those that inhabit different regions and ecosystems. In the worst case scenario, the Oromo in Bale may have already lost much of the plant knowledge that previous generations relied on for centuries.

## Competing interests

The authors declare that they have no competing interests.

## Authors' contributions

RB and PS collected/identified plant material under the voucher acronym RBU. RB, PS and AW conducted the interview work. RB analyzed the data and wrote the manuscript. PE elaborated on the Figures and the site description, and conducted the statistical analysis of the data as well as writing the manuscript. All authors have read and approved the final manuscript.
